# Impact of Frailty and Diaphragmatic Dysfunction on Outcomes in ARDS: Focusing on Perioperative Respiratory Management

**DOI:** 10.1111/crj.70213

**Published:** 2026-07-08

**Authors:** Mengmei Ma, Lianchao Men, Chenhui Fu, Bin Huang, Yu Han, Danyang Wang

**Affiliations:** ^1^ Department of Critical Care Medicine Hejian Branch of Cangzhou Central Hospital CangZhou Hebei China; ^2^ Department of Emergency Medicine Hejian Branch of Cangzhou Central Hospital CangZhou Hebei China; ^3^ Department of Ultrasound Medical Center Cangzhou Central Hospital CangZhou Hebei China; ^4^ Department of Dean Office Cangzhou Central Hospital CangZhou Hebei China; ^5^ Department of Ultrasound Hejian Branch of Cangzhou Central Hospital CangZhou City Hebei China

**Keywords:** acute respiratory distress syndrome, clinical outcomes, diaphragm ultrasound, diaphragmatic dysfunction, frailty, perioperative respiratory management

## Abstract

**Objective:**

The objective of this study is to assess the independent and combined impact of frailty (measured by the Clinical Frailty Scale, CFS) and diaphragmatic dysfunction (measured by diaphragm thickening fraction, DTF) on clinical outcomes in patients with acute respiratory distress syndrome (ARDS) and to discover if perioperative respiratory management strategies moderate these relationships.

**Methods:**

A single‐center retrospective cohort study of ARDS patients with radiographic severity classification who visited the ICU of Cangzhou Central Hospital between January 2020 and December 2023 was performed. Patients were divided in a frail group (CFS ≥ 4, *n* = 87) and nonfrail group (CFS < 4, *n* = 124). Within 24 h of ICU admission and on Days 3 and 7 of mechanical ventilation, bedside diaphragm ultrasound was performed; DTF and diaphragm excursion (DE) were measured. Outcome measures included mechanical ventilation parameters, perioperative respiratory management strategies, and clinical outcomes (28‐ and 90‐day mortality, duration of mechanical ventilation, and ICU length of stay). Independent and interactive effects of frailty and diaphragmatic dysfunction on outcomes were evaluated using multivariable logistic regression, Cox proportional hazards models, and interaction analysis.

**Results:**

Frail patients were significantly older (71.3 vs. 58.4 years; *p* < 0.001) and had higher APACHE II and SOFA scores. Dynamic pulmonary function at rest was compared between the two groups; the frailty group demonstrated significantly lower DTF and higher proportions with diaphragmatic dysfunction (DTF < 20%: 16.2% ± 8.4% [vs. 28.7% ± 9.6%, *p* < 0.001] and DTF < 20%). Frail patients had significantly higher rates of 28‐day mortality (44.8% vs. 23.4%, *p* = 0.002) and 90‐day mortality (59.8% vs. 30.6%, *p* < 0.001). CFS ≥ 4 (OR = 2.84, 95% CI: 1.61–5.02) and DTF < 20% (OR = 3.16, 95% CI: 1.77–5.63) remained independently associated with mortality at Day 28 in multivariable analysis; the interaction term generated an even greater risk for mortality to occur (OR = 4.23, 95% CI: 2.37–7.54). In subgroup analysis, 28‐day mortality was 55.6% for patients with frailty and concurrent diaphragmatic dysfunction. The combined predictive model including the CFS, DTF, and APACHE II scores was found to have an AUC of 0.851. In frail patients, mechanical ventilation duration was shorter in those who received inspiratory muscle training and early rehabilitation than in controls. In a 1:1 propensity score–matched sensitivity analysis (*n* = 148), both frailty (OR = 2.61, 95% CI: 1.42–4.79) and DTF < 20% (OR = 2.94, 95% CI: 1.58–5.47) were unchanging independent predictors; additive interaction was confirmed, RERI = 1.94, AP = 0.46, SI = 2.31. After IPTW adjustment, the IMT‐treated frail patients had a 3.1‐day (95% CI: 0.4–5.8, *p* = 0.024) reduction in mechanical ventilation duration with a nonsignificant trend toward lower 28‐day mortality (*p* = 0.198).

**Conclusions:**

Frailty and diaphragmatic dysfunction both have independent adverse effects on outcomes in ARDS, as well as a synergistic effect. However, systematic perioperative diaphragm ultrasound monitoring, personalized lung‐protective ventilation strategies, and early inspiratory muscle rehabilitation have the potential to improve clinical outcomes in frail patients with concomitant disordered diaphragmatic function.

## Graphical abstract

This study demonstrates that frailty and diaphragmatic dysfunction independently and synergistically increase mortality risk in ARDS patients, with concurrent conditions amplifying 28‐day mortality to 55.6%. A combined CFS‐DTF‐APACHE II predictive model (AUC = 0.851–0.889) effectively identifies high‐risk patients, supporting routine integration of frailty assessment and diaphragm ultrasound monitoring into perioperative respiratory management to guide individualized interventions including inspiratory muscle training and early rehabilitation.

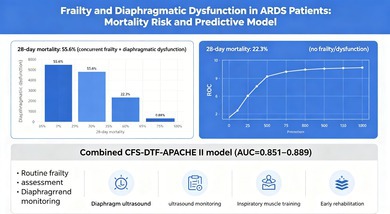

## 1 Introduction

Acute respiratory distress syndrome (ARDS) is among the most difficult critical care complications, with refractory hypoxemia, decreased lung compliance, and bilateral diffuse pulmonary infiltrates. The large multicenter international LUNG SAFE study estimates that 10.4% of patients admitted to ICU have ARDS, with a 28‐day mortality in excess of 40.3%, and further exceeding 46% in severe cases [1]. Over the past decade, the clinical course of ARDS has been markedly improved due to widespread adoption of lung‐protective ventilation strategies [1]. However, overall mortality remains unacceptably high, and long‐term functional impairment among survivors adds an additional burden to the clinical community [2].

Skeletal muscle dysfunction as a central aspect of the pathophysiology of ARDS has been increasingly recognized in recent years. The diaphragm is the main respiratory muscle [3] and encounters multiple injury mechanisms in ARDS: Ventilator‐induced diaphragmatic dysfunction (VIDD) due to disuse atrophy occurs within 18–69 h of initiation of mechanical ventilation [4], whereas systemic inflammation arising from sepsis, malnutrition, and exposure to neuromuscular blocking agents may further contribute to diaphragmatic injury [5, 6]. In particular, diaphragm weakness and limb muscle weakness commonly co‐exist but are separate diseases with individual prognostic effect on weaning failure [7]. Bedside ultrasound has been gradually established as the new noninvasive gold standard for determination of outward diaphragmatic dysfunction, but also offers a noninvasive and reproducible assessment method that can provide real‐time information on diaphragm thickness and contractile function [8, 9, 10].

Simultaneously, frailty—an age‐related syndrome associated with decreased physiological reserve across multiple organ systems—has been identified as an independent predictor of outcomes in patients admitted to the ICU [11, 12]. The CFS can be easily administered, has proven validity and reliability, and is ideally designed for the rapid assessment of frailty among ICU patients [13]. Frail patients are vulnerable to acute critical illness due to loss of muscle reserve, diminished immunoregulatory capacity, and inadequate nutritional substrate causing a rapid decline in diaphragmatic function, which may start an accelerating death spiral of respiratory muscle impairment and worsening frailty. But, systematic studies pursuing the effects of frailty severity and concomitant diaphragmatic dysfunction in relation to ARDS end result measures, and the capacity mediation or moderation of perioperative respiration control techniques proceed to be missing.

This is especially important with regard to the perioperative respiratory management in patients suffering from ARDS. Patients with ARDS who underwent postoperative surgery are characteristically at a much greater need for mechanical ventilation dependency than nonsurgical patients because of surgical stress but also simply anaesthetic residual effects and possibly compromised diaphragm function as well. In theory, individualized lung‐protective ventilation strategies, inspiratory muscle training (IMT), and early rehabilitation are able to oppose the detrimental effects of frailty via enhancement of diaphragmatic function; however, their clinical efficacy—especially net survival benefit—is not consistently confirmed in studies with large samples [14, 15, 16].

Frailty and diaphragmatic dysfunction are two patient factors that play a synergistic role in determining prognosis, mortality risk stratification, and treatment management of ARDS patients following ICU admission, but potential underlying interactions between respiratory management including airway pressure release ventilation (APRV) methods have not been fully explored. This study conducted a retrospective examination of clinical data from 211 ARDS patients who were admitted to the intensive care unit (ICU) at Cangzhou Central Hospital in China to systematically evaluate the univariate main effects and bivariate synergistic effects of frailty status on clinical outcome as well as the effect of integrated perioperative respiratory management strategies on respiratory dynamic outcomes among this patient population—thereby providing evidence‐based support for behavioral therapy‐based specific regimens for individualized ARDS treatment.

## 2 Materials and Methods

### 2.1 Study Design and Patient Enrollment

This single‐center retrospective cohort study was conducted from January 2020 to December 2023 at the ICU of Cangzhou Central Hospital. The study protocol was approved by the Institutional Review Board of Cangzhou Central Hospital (IRB reference: 2024‐315‐02). Inclusion criteria were as follows: (1) age ≥ 18 years; (2) ARDS diagnosis meeting the Berlin criteria (onset within 1 week, bilateral pulmonary infiltrates, noncardiogenic pulmonary edema, and PaO_2_/FiO_2_ < 300 mmHg with PEEP ≥ 5 cmH_2_O); (3) completion of CFS assessment and diaphragm ultrasound within 24 h of ICU admission; (4) invasive mechanical ventilation duration ≥ 48 h; and (5) complete clinical data. Exclusion criteria were as follows: (1) pre‐existing neuromuscular disease (including myasthenia gravis and amyotrophic lateral sclerosis); (2) history of diaphragmatic paralysis or phrenic nerve injury; (3) pregnancy or lactation; (4) refusal to participate (by family proxy); and (5) inadequate ultrasound image quality precluding accurate diaphragm parameter measurement. Sepsis‐related ARDS etiology was defined using the Sepsis‐3 consensus criteria [17].

### 2.2 Frailty Assessment

All patients underwent CFS assessment within 24 h of ICU admission by a trained ICU nurse or physician. The CFS is a 9‐point scale (scores 1–9), with scores 1–3 indicating *nonfrail*, 4–6 indicating *mild‐to‐moderate frailty*, and 7–9 indicating *severe frailty* [13]. In this study, a CFS score ≥ 4 was used as the threshold for defining frailty. For patients unable to complete an interview, a primary caregiver provided proxy responses based on functional status in the 2 weeks prior to hospital admission. In this cohort, proxy assessments were completed for 38 of 87 frail patients (43.7%) and 12 of 124 nonfrail patients (9.7%). Inter‐rater reliability was evaluated in a randomly selected 30% subsample (*n* = 63) with independently scored duplicate assessments; the intraclass correlation coefficient (ICC) between trained raters was 0.84 (95% CI: 0.76–0.90), indicating good reliability. Proxy‐based underestimation of frailty severity is acknowledged as a nondifferential misclassification error biasing associations toward the null.

### 2.3 Diaphragm Ultrasound Assessment

Diaphragm ultrasound was performed at baseline (within 24 h of ICU admission), on Day 3, and on Day 7 of mechanical ventilation by standardized‐trained operators blinded to clinical data. Using a 7.5‐MHz high‐frequency linear array transducer placed in the right mid‐axillary line at the 8th–9th intercostal space, end‐expiratory (Tee) and end‐inspiratory (Tei) diaphragm thickness were measured in B‐mode, and the diaphragm thickening fraction (DTF) was calculated as DTF = (Tei − Tee)/Tee × 100% [8]. Diaphragm excursion (DE) was additionally measured in M‐mode via the subcostal window. A DTF < 20% was used to define diaphragmatic dysfunction. All measurements represent the average of three consecutive respiratory cycles.

### 2.4 Perioperative Respiratory Management

Mechanical ventilation followed both the ARDSNet lung‐protective ventilation protocol and emerging lung–diaphragm protective principles [18]: tidal volume of 6‐mL/kg predicted body weight (PBW), plateau pressure < 30 cmH_2_O, and individualized PEEP titration (using the FiO_2_–PEEP table or esophageal pressure measurement) [19]. Prone positioning (12 h/day) was considered for severe ARDS (PaO_2_/FiO_2_ < 150 mmHg). Spontaneous breathing trials (SBT) were conducted using a T‐piece or low‐level pressure support ventilation, with the SBT pass criteria in accordance with the 2017 ATS/ESICM/SCCM ARDS clinical practice guideline [19].

IMT protocol: In hemodynamically stable patients (FiO_2_ < 0.6, PEEP < 10 cmH_2_O) who were conscious and cooperative, respiratory therapists administered IMT twice daily, 15 min per session, at a load of 30%–40% of maximal inspiratory pressure (PImax), with progressive load increases every 3 days based on tolerance [20]. Early rehabilitation was conducted by physical therapists, encompassing passive range‐of‐motion exercises, active‐assisted movements, and sitting‐up training, initiated within 48 h of hemodynamic stabilization.

### 2.5 Primary and Secondary Outcomes

The primary outcome was all‐cause 28‐day mortality. Secondary outcomes included 90‐day mortality, total duration of mechanical ventilation, ICU length of stay, total hospital length of stay, weaning failure rate (requirement for reintubation or NIV failure within 48 h after extubation), incidence of ICU‐acquired weakness (MRC muscle strength sum score < 48), and rate of hospital‐acquired infections. Specifically, weaning failure is defined as failure to tolerate the SBT or requirement for noninvasive ventilation within 48 h of planned extubation; reintubation refers exclusively to endotracheal re‐intubation within 48 h and is reported as a separate, nonoverlapping outcome. ICU‐acquired weakness (MRC sum score < 48) was assessed on the first day on which the patient was sufficiently alert (RASS ≥ −1) and cooperative to complete the full 12‐muscle‐group MRC examination, confirmed on two consecutive assessments. Throughout the manuscript, hazard ratios (HR) are reported exclusively for Cox proportional hazards models and odds ratios (OR) exclusively for logistic regression models, with model type labeled in each table footnote.

### 2.6 Statistical Analysis

Continuous variables are expressed as mean ± SD or median (IQR) and compared by *t*‐test or Mann–Whitney U test; categorical variables as frequency (%) by chi‐square or Fisher's exact test. Multivariable logistic regression (backward stepwise, *p* < 0.1 entry) yielded ORs with 95% CIs. Survival was compared by Kaplan–Meier/log‐rank; model discrimination by ROC/AUC (DeLong pairwise comparisons). Frailty–diaphragm interaction was assessed multiplicatively and additively (RERI, AP, SI; R package “epiR”). Confounding was addressed by 1:1 PSM and IPTW. Subgroup comparisons were corrected by Benjamini–Hochberg FDR (q < 0.05). Calibration was assessed by Hosmer–Lemeshow test with bootstrap internal validation. All analyses used R 4.3.2 (two‐sided *p* < 0.05).

## 3 Results

### 3.1 Baseline Characteristics

From 347 patients screened, 211patients per protocol were enrolled (frailty group, *n* = 87; nonfrailty group, *n* = 124). Clinical characteristics at baseline are presented in Table 1. Frail patients were significantly older (71.3 ± 9.2 vs. 58.4 ± 11.7 years), had lower BMI (21.4 ± 3.6 vs. 24.2 ± 4.1 kg/m^2^), and higher prevalences of hypertension (70.1% vs. 50.0%) and diabetes mellitus (49.4% vs. 30.6%) (all *p* < 0.01). There were no differences between groups in sex distribution and ARDS etiology (*p* > 0.05). The frailty group had more severe disease, with higher APACHE II (26.3 ± 7.1 vs. 21.4 ± 6.8) and SOFA scores (9.8 ± 3.2 vs. 7.6 ± 2.9) (*p* < 0.001).

**TABLE 1:** Baseline clinical characteristics (*n* = 211).

Variable	Frailty group (*n* = 87)	Nonfrailty group (*n* = 124)	All patients (*n* = 211)	*p*
Age (years)	71.3 ± 9.2	58.4 ± 11.7	63.6 ± 12.8	< 0.001
Male sex, *n* (%)	52 (59.8)	74 (59.7)	126 (59.7)	0.987
BMI (kg/m^2^)	21.4 ± 3.6	24.2 ± 4.1	23.1 ± 4.1	< 0.001
APACHE II score	26.3 ± 7.1	21.4 ± 6.8	23.4 ± 7.3	< 0.001
SOFA score	9.8 ± 3.2	7.6 ± 2.9	8.5 ± 3.2	< 0.001
CFS score (median)	5 (4–6)	2 (1–3)	3 (2–5)	< 0.001
Hypertension, *n* (%)	61 (70.1)	62 (50.0)	123 (58.3)	0.006
Diabetes mellitus, *n* (%)	43 (49.4)	38 (30.6)	81 (38.4)	0.008
Chronic kidney disease, *n* (%)	28 (32.2)	19 (15.3)	47 (22.3)	0.005
Malignancy, *n* (%)	22 (25.3)	18 (14.5)	40 (19.0)	0.054
ICU admission cause—pneumonia, *n* (%)	54 (62.1)	71 (57.3)	125 (59.2)	0.502
ICU admission cause—sepsis, *n* (%)	21 (24.1)	33 (26.6)	54 (25.6)	0.683
ICU admission cause—postoperative, *n* (%)	12 (13.8)	20 (16.1)	32 (15.2)	0.646
PaO_2_/FiO_2_ (mmHg)	138.6 ± 42.4	161.2 ± 51.8	151.9 ± 49.8	0.001

*Note:* Data are presented as mean ± SD or *n* (%).

Abbreviations: APACHE II, Acute Physiology and Chronic Health Evaluation II score; BMI, body mass index; CFS, Clinical Frailty Scale; PaO_2_/FiO_2_, arterial partial pressure of oxygen to fraction of inspired oxygen ratio; SOFA, Sequential Organ Failure Assessment score.

### 3.2 Diaphragm Ultrasound Parameters

Ultrasound parameters for diaphragm ultrasound are shown in Table 2. Diaphragm function parameters were significantly lower in the frailty group than in the nonfrailty group, including baseline DTF (16.2% ± 8.4% vs. 28.7% ± 9.6%, *p* < 0.001), end‐expiratory thickness (1.9 ± 0.6 vs. 2.4 ± 0.7 mm, *p* < 0.001), end‐inspiratory thickness (2.2 ± 0.7 vs. 3.4 ± 0.8 mm, *p* < 0.001), and DE (9.4 ± 4.1 vs. 18.6 ± 5.3 mm, all *p* < 0.001). Diaphragmatic dysfunction (DTF < 20%) was more common in frail patients (72.4% vs. 33.1%, *p* < 0.001), as were bilateral muscle dysfunction (56.3% vs. 20.2%, *p* < 0.001). The serial monitoring revealed a progressive decline of DTF in both groups, more pronounced in the frailty group (Day 3: 14.1% ± 7.2%; Day 7: 12.3% ± 6.8%).

**TABLE 2:** Comparison of diaphragm ultrasound parameters.

Ultrasound parameter	Frailty group (*n* = 87)	Nonfrailty group (*n* = 124)	*p*	Reference range
DTF (%)	16.2 ± 8.4	28.7 ± 9.6	< 0.001	> 20%
End‐expiratory thickness (Tee, mm)	1.9 ± 0.6	2.4 ± 0.7	< 0.001	2.0–4.0
End‐inspiratory thickness (Tei, mm)	2.2 ± 0.7	3.4 ± 0.8	< 0.001	3.0–5.5
Diaphragm excursion (mm)	9.4 ± 4.1	18.6 ± 5.3	< 0.001	> 15
VIDD incidence, *n* (%)	63 (72.4)	41 (33.1)	< 0.001	—
Diaphragm atrophy, *n* (%)	38 (43.7)	12 (9.7)	< 0.001	—
Bilateral diaphragm dysfunction, *n* (%)	49 (56.3)	25 (20.2)	< 0.001	—
DTF on MV Day 3 (%)	14.1 ± 7.2	25.3 ± 8.4	< 0.001	> 20%
DTF on MV Day 7 (%)	12.3 ± 6.8	22.8 ± 8.1	< 0.001	> 20%

*Note:* Data are presented as mean ± SD or *n* (%).

Abbreviations: DTF, diaphragm thickening fraction; MV, mechanical ventilation; Tee, end‐expiratory diaphragm thickness; Tei, end‐inspiratory diaphragm thickness; VIDD, ventilator‐induced diaphragmatic dysfunction.

### 3.3 Mechanical Ventilation Parameters

Table 3 presents the mechanical ventilation parameters. Tidal volumes were comparable between groups (6.3 ± 0.8 vs. 6.4 ± 0.7‐mL/kg PBW; *p* = 0.354). Plateau pressures (28.4 ± 4.6 vs. 25.3 ± 4.1 cmH_2_O), driving pressures (16.8 ± 4.2 vs. 14.1 ± 3.8 cmH_2_O), mechanical power (18.6 ± 5.3 vs. 14.2 ± 4.1 J/min), and P0 1 (2.8 ± 1.2 vs. 1.9 ± 0.9 cmH_2_O) (all *p* < 0.001). The rates of prone position were similar (59.8% vs. 46.8%, *p* = 0.077), but the success of SBT was significantly lower in frail patients (32.2% vs. 67.7%, *p* < 0.001).

**TABLE 3:** Comparison of mechanical ventilation parameters.

Ventilation parameter	Frailty group (*n* = 87)	Nonfrailty group (*n* = 124)	All patients (*n* = 211)	*p*
Tidal volume (mL/kg PBW)	6.3 ± 0.8	6.4 ± 0.7	6.4 ± 0.8	0.354
PEEP (cmH_2_O)	8.6 ± 2.4	7.8 ± 2.1	8.1 ± 2.3	0.018
Plateau pressure (cmH_2_O)	28.4 ± 4.6	25.3 ± 4.1	26.6 ± 4.5	< 0.001
Driving pressure (cmH_2_O)	16.8 ± 4.2	14.1 ± 3.8	15.2 ± 4.1	< 0.001
P0.1 (cmH_2_O)	2.8 ± 1.2	1.9 ± 0.9	2.3 ± 1.1	< 0.001
Mechanical power (J/min)	18.6 ± 5.3	14.2 ± 4.1	16.0 ± 5.1	< 0.001
Prone positioning, *n* (%)	52 (59.8)	58 (46.8)	110 (52.1)	0.077
Neuromuscular blockade, *n* (%)	43 (49.4)	41 (33.1)	84 (39.8)	0.020
Successful SBT, *n* (%)	28 (32.2)	84 (67.7)	112 (53.1)	< 0.001

*Note:* Data are presented as mean ± SD or *n* (%).

Abbreviations: P0.1: airway occlusion pressure (respiratory drive indicator); PBW: predicted body weight; PEEP: positive end‐expiratory pressure; SBT: spontaneous breathing trial.

### 3.4 Clinical Outcomes

Comparison of clinical outcomes is in Table 4. The frail patients had dramatically longer ICU stay (18.4 ± 9.6 vs. 11.2 ± 6.3 days), mechanical ventilation duration (14.6 ± 8.1 vs. 8.3 ± 5.4 days), and total hospital stay (28.7 ± 12.4 vs. 19.6 ± 9.8 days) (all *p* < 0.001). The frailty group had significantly higher rates of weaning failure (54.0% vs. 22.6%), reintubation (35.6% vs. 12.9%), tracheostomy (43.7% vs. 17.7%), and ICU‐acquired weakness (66.7% vs. 31.5%) (*p* < 0.001). Frail patients had increased 28‐day mortality (44.8% vs. 23.4%; *p* = 0.002) and 90‐day mortality (59.8% vs. 30.6%; *p* < 0.001).

**TABLE 4:** Comparison of clinical outcomes.

Clinical outcome	Frailty group (*n* = 87)	Nonfrailty group (*n* = 124)	All patients (*n* = 211)	*p*
ICU LOS (days)	18.4 ± 9.6	11.2 ± 6.3	14.2 ± 8.7	< 0.001
MV duration (days)	14.6 ± 8.1	8.3 ± 5.4	10.9 ± 7.3	< 0.001
Total hospital stay (days)	28.7 ± 12.4	19.6 ± 9.8	23.4 ± 11.8	< 0.001
Weaning failure, *n* (%)	47 (54.0)	28 (22.6)	75 (35.5)	< 0.001
Reintubation, *n* (%)	31 (35.6)	16 (12.9)	47 (22.3)	< 0.001
Tracheostomy, *n* (%)	38 (43.7)	22 (17.7)	60 (28.4)	< 0.001
ICU‐acquired weakness, *n* (%)	58 (66.7)	39 (31.5)	97 (46.0)	< 0.001
28‐day mortality, *n* (%)	39 (44.8)	29 (23.4)	68 (32.2)	0.002
90‐day mortality, *n* (%)	52 (59.8)	38 (30.6)	90 (42.7)	< 0.001
Hospital‐acquired infection, *n* (%)	42 (48.3)	38 (30.6)	80 (37.9)	0.012

*Note:* ICU‐acquired weakness defined as MRC muscle strength sum score < 48. Data are presented as mean ± SD or *n* (%).

Abbreviations: ICU, intensive care unit; LOS, length of stay; MV, mechanical ventilation.

### 3.5 Independent and Synergistic Effects of Frailty and Diaphragmatic Dysfunction on Mortality

After multivariable adjustment, frailty (OR = 2.84, 95% CI: 1.61–5.02) and diaphragmatic dysfunction (OR = 3.16, 95% CI: 1.77–5.63) both remained independent predictors of 28‐day mortality (both *p* < 0.001; Table 5). The interaction term for frailty and the diaphragm was highly significant (OR = 4.23, 95% CI: 2.37–7.54, *p* < 0.001), which further demonstrated supra‐additive synergy confirmed by additive interaction metrics (RERI = 1.94, AP = 0.46, SI = 2.31; all *p* < 0.05). The sensitivity analysis for PSM (*n* = 148) demonstrating a similar pattern: frailty OR = 2.61; DTF < 20% OR = 2.94; interaction OR = 3.87; all *p* < 0.001. All of these additive interaction metrics are reported in the revised Table 5.

**TABLE 5:** Multivariable logistic regression analysis for 28‐day mortality.

Variable	OR	SE	95% CI lower	95% CI upper	*p*
CFS ≥ 4 (frailty)	2.84	0.63	1.61	5.02	< 0.001
DTF < 20% (diaphragm dysfunction)	3.16	0.71	1.77	5.63	< 0.001
Age (per 10‐year increment)	1.48	0.29	1.02	2.14	0.038
APACHE II score	1.12	0.04	1.05	1.20	< 0.001
PaO_2_/FiO_2_ < 100 mmHg	2.31	0.54	1.32	4.04	0.003
Driving pressure > 15 cmH_2_O	1.94	0.47	1.11	3.38	0.019
Bilateral diaphragm dysfunction	2.07	0.49	1.19	3.61	0.010
Mechanical power > 17 J/min	1.76	0.43	1.03	3.01	0.039
Frailty × diaphragm dysfunction (interaction)	4.23	1.12	2.37	7.54	< 0.001
RERI (additive interaction)	1.94	—	0.63	3.25	0.012
Attributable proportion (AP)	0.46	—	0.18	0.74	0.008
Synergy index (SI)	2.31	—	1.28	4.17	0.006

*Note:* All models passed the Hosmer–Lemeshow goodness‐of‐fit test (*p* > 0.05).

Abbreviations: CFS, Clinical Frailty Scale; CI, confidence interval; DTF, diaphragm thickening fraction; OR, odds ratio; P/F, PaO_2_/FiO_2_ ratio; SE, standard error.

### 3.6 Subgroup Analysis

Subgroup analysis (Table 6) revealed that the relative to nonfrail/nondysfunctional patients (*n* = 83, 28‐day mortality 16.9%) mortality increased successively: nonfrail + dysfunctional (31.7%; HR = 1.89), frail + normal diaphragm function (33.3%; HR = 2.11), and frail + diaphragmatic dysfunction (55.6%; HR = 4.08; 95% CI: 2.32–7.17); *p* < 0.001. Synergism was greatest in elderly (≥ 65 years) and severe ARDS (PaO_2_/FiO_2_ < 100 mmHg) subgroups; the postoperative subgroup did not achieve significance (*p* = 0.148) and is hypothesis‐generating only.

**TABLE 6:** Subgroup analysis by frailty–diaphragmatic dysfunction combination.

Subgroup	*n*	28‐day mortality	HR	95% CI	*p*
Nonfrail + normal diaphragm	83	16.9%	Reference	—	—
Nonfrail +diaphragm dysfunction	41	31.7%	1.89	1.02–3.49	0.042
Frail + normal diaphragm	24	33.3%	2.11	1.04–4.28	0.038
Frail +diaphragm dysfunction	63	55.6%	4.08	2.32–7.17	< 0.001
Elderly subgroup (≥ 65 years)	94	47.9%	3.24	1.98–5.31	< 0.001
Severe ARDS (P/F < 100)	68	52.9%	3.87	2.19–6.84	< 0.001
Postoperative ARDS	32	25.0%	1.76	0.82–3.77	0.148
Sepsis‐associated ARDS	54	46.3%	3.12	1.74–5.58	< 0.001

*Note:* Reference group: nonfrail + normal diaphragm (*n* = 83). Cox proportional hazards model adjusted for age, APACHE II score, and PaO_2_/FiO_2_.

Abbreviations: CI, confidence interval; HR, hazard ratio.

### 3.7 Perioperative Respiratory Management Strategies

Table 7 shows the distribution of perioperative respiratory management interventions. Early rehabilitation and IMT were less frequently performed in frail patients (33.3% vs. 58.1%, *p* = 0.001; 27.6% vs. 42.7%, *p* = 0.034). In frail patients receiving IMT and early rehabilitation, mechanical ventilation duration was shorter (11.3 vs. 15.5 days, *p* = 0.038); this difference was 3.1 days (95% CI: 0.4–5.8, *p* = 0.024) after IPTW adjustment with a nonsignificant mortality trend (*p* = 0.198). This finding is considered exploratory.

**TABLE 7:** Distribution of perioperative respiratory management interventions.

Perioperative respiratory management	Frailty group (*n* = 87)	Nonfrailty group (*n* = 124)	*p*	Recommendation grade
Preoperative pulmonary function testing, *n* (%)	61 (70.1)	106 (85.5)	0.011	Grade I
Prophylactic CPAP, *n* (%)	18 (20.7)	22 (17.7)	0.597	Grade IIa
Intraoperative lung‐protective ventilation, *n* (%)	74 (85.1)	114 (91.9)	0.128	Grade I
Intraoperative PEEP optimization, *n* (%)	51 (58.6)	89 (71.8)	0.057	Grade IIa
Early postoperative rehabilitation, *n* (%)	29 (33.3)	72 (58.1)	0.001	Grade I
Diaphragm ultrasound monitoring, *n* (%)	87 (100.0)	124 (100.0)	—	Grade IIa
Inspiratory muscle training (IMT), *n* (%)	24 (27.6)	53 (42.7)	0.034	Grade IIb
Postural drainage, *n* (%)	38 (43.7)	41 (33.1)	0.131	Grade IIa
Nutritional support optimization, *n* (%)	72 (82.8)	101 (81.5)	0.818	Grade I
Multidisciplinary consultation, *n* (%)	58 (66.7)	62 (50.0)	0.022	Grade IIa

*Note:* Recommendation grades derived from the 2017 ARDS clinical practice guidelines and the ABCDE bundle. Data are presented as *n* (%).

Abbreviations: CPAP, continuous positive airway pressure; IMT, inspiratory muscle training; PEEP, positive end‐expiratory pressure.

### 3.8 Predictive Model Performance

The findings of the various discriminative models are summarized in Table 8. The discriminative power of individual predictors was modest (CFS AUC = 0.712; DTF AUC = 0.741; APACHE II AUC = 0.723). The combined CFS + DTF model resulted in AUC = 0.814, which increased to 0.851 with APACHE II and 0.889 for the full model (Bootstrap‐validated AUC = 0.873). Calibration was good (Hosmer–Lemeshow *p* = 0.45), at the optimal cutoff (threshold: 0.42) PPV= 71.8% and NPV = 87.6%.

**TABLE 8:** Comparison of discriminative performance among different predictive models.

Predictive model	AUC	Sensitivity (%)	Specificity (%)	Youden index	Brier score
CFS alone	0.712	72.1	66.3	0.384	0.198
DTF alone	0.741	75.0	68.5	0.435	0.187
APACHE II alone	0.723	70.6	67.8	0.384	0.193
CFS + DTF combined	0.814	80.9	74.2	0.551	0.162
CFS + DTF + APACHE II	0.851	84.1	76.6	0.607	0.148
Full model (all significant variables)	0.889	87.3	80.1	0.674	0.131
Internal validation (bootstrap)	0.873	85.6	78.4	0.640	0.139
Calibration (H‐L χ^2^ = 7.84, *p* = 0.45)	—	—	—	—	—
Optimal cutoff (threshold: 0.42)	—	87.3	80.1	0.674	—
PPV/NPV at optimal cutoff	PPV = 71.8%; NPV = 87.6%	—	—	—	—

*Note:* Bootstrap internal validation performed with 1000 replications. A lower Brier score indicates higher predictive accuracy. Youden Index = Sensitivity + Specificity − 1.

Abbreviation: AUC, area under the receiver operating characteristic curve.

### 3.9 Survival Analysis and ROC Curves

**FIGURE 1:**
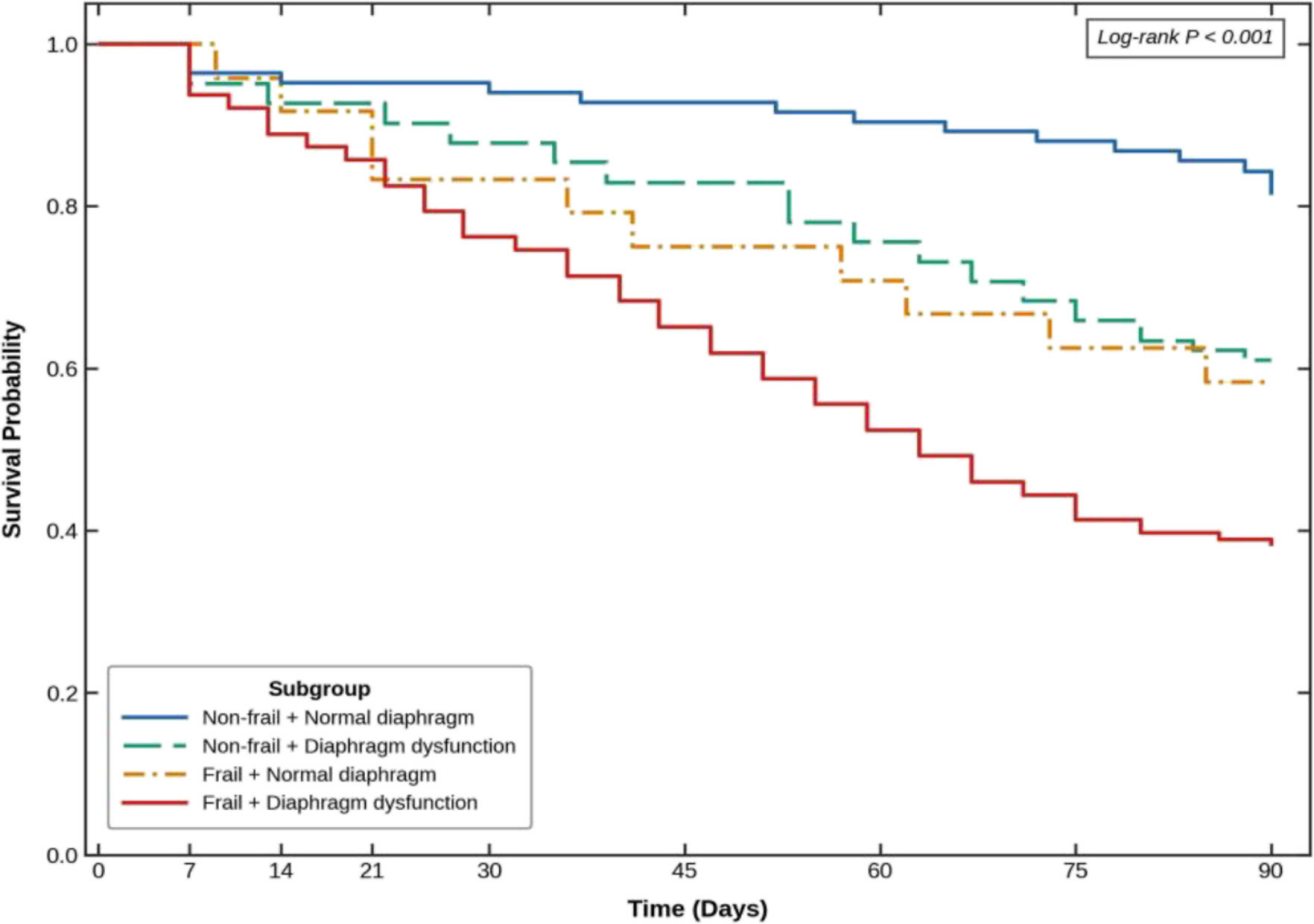
Kaplan–Meier survival curves for the four frailty–diaphragmatic dysfunction subgroups over 90‐day follow‐up. The frail + diaphragmatic dysfunction group (red solid line) had the lowest survival rate, whereas the nonfrail + normal diaphragm group (blue dashed line) had the highest. Differences among groups were statistically significant (log‐rank *p* < 0.001). CFS, Clinical Frailty Scale; DTF, diaphragm thickening fraction.

**FIGURE 2:**
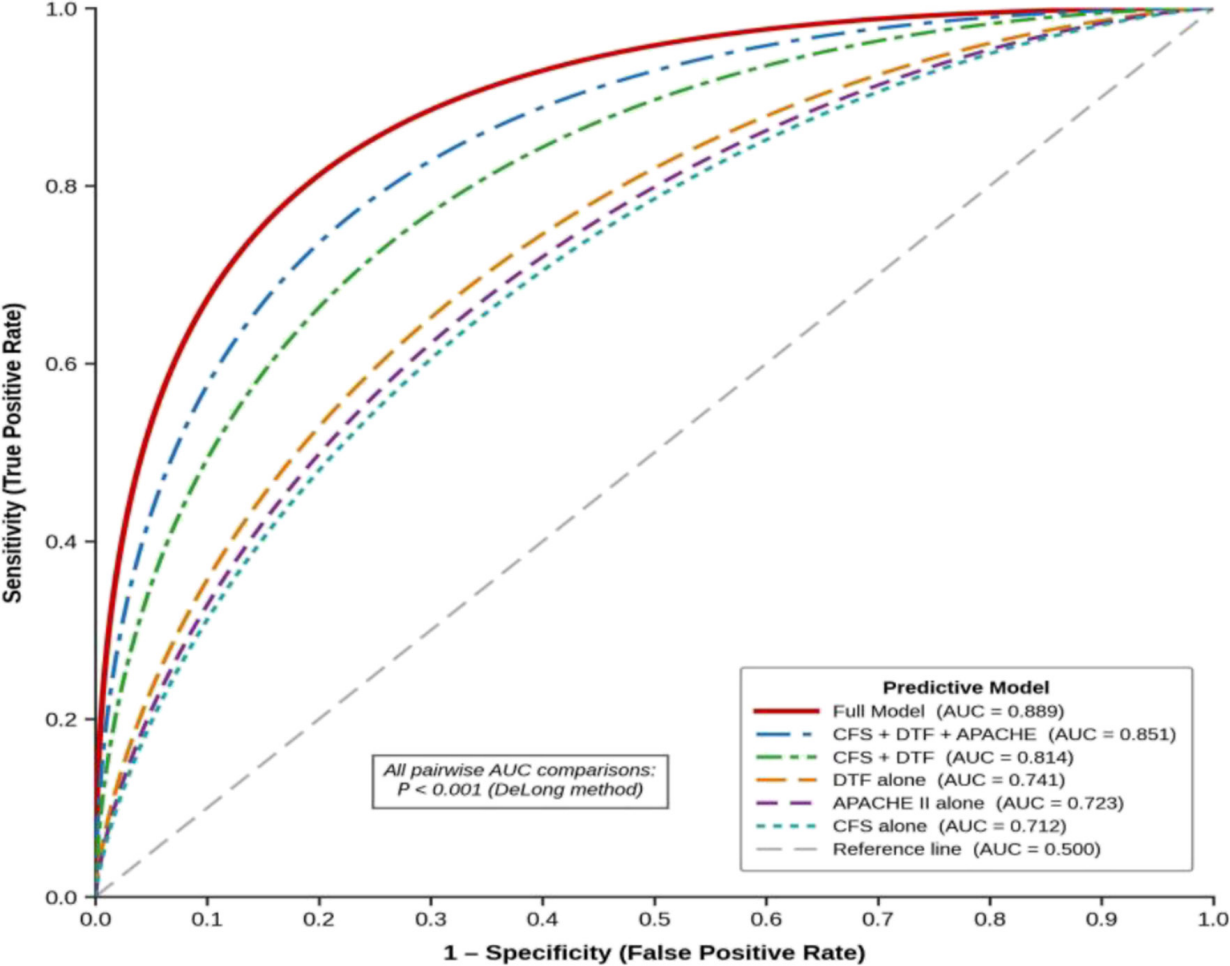
Receiver operating characteristic (ROC) curve comparison for different predictive models. The full predictive model (AUC = 0.889, black solid line) significantly outperformed individual indicators: CFS alone (AUC = 0.712, green dashed line), DTF alone (AUC = 0.741, blue dotted line), and APACHE II alone (AUC = 0.723, red dash‐dot line). All pairwise AUC differences were statistically significant (*p* < 0.001, DeLong method).

Kaplan–Meier curves at 90‐day follow‐up for the four subgroups are displayed in Figure 1. The higher 90‐day survival of the frail + normal diaphragm group compared to the frail + diaphragmatic dysfunction group was statistically significant (81.4% vs. 38.1%, log‐rank *p* < 0.001). The survival curves of the two intermediate subgroups crossed previously but separated after Day 30. ROC curves for the different predictive models are shown in Figure 2; there was a significant improvement of the full predictive model (AUC = 0.889) over any individual indicator (*p* < 0.001).

## 4 Discussion

This appears the first single‐center, retrospective clinical study to systematically assess the combined impact of frailty severity and diaphragmatic dysfunction on clinical outcomes in ARDS. The main importance of our experiment is fourfold: Firstly, both frailty and diaphragmatic dysfunction are independent risk factors for 28‐day mortality in patients with ARDS; secondly, the two conditions exert strong synergistic effects on prognosis, where concurrent frailty and diaphragmatic dysfunction confer a significant mortality risk (HR = 4.08 by Cox subgroup analyses, Table 6); logistic interaction OR = 4.23 (Table 5); additive supra‐additivity was also confirmed by RERIs of 1.94 between cohorts, AP score values of 0.46 as the product term UP‐β × DP‐β (high scores vs. low) and SI values of > 2.31 to indicate supra‐additivity); thirdly a combined predictive model incorporating CFS/Dyspnoea Scale APACHE II scores has better discrimination than any single indicator (AUC: 0.851–0.889); finally, valid strategies for effective perioperative respiratory management especially IMT therapy or early rehabilitation had favorable impact on weaning success rates/outcomes in frail patients.

The incidence of diaphragmatic dysfunction in this cohort of ARDS was high (49.8% for all patients, 105/211), and in keeping with the wide variation reported in prior literature (33%–80%) [21]. Diaphragmatic dysfunction was significantly more common in frail (72.4%) than in nonfrail patients (33.1%); the pathophysiological mechanisms responsible are multifactorial [7]. First, compared to healthy controls, frail patients have lower baseline muscle mass with reduced myofiber cross‐sectional area and increased susceptibility to ventilator‐induced atrophy from a reduction in type I slow‐twitch fibers along with a shift toward type II fast‐twitch fibers that also increase the diaphragm susceptibility [4, 5, 22]. Second, the chronic inflammatory status associated with frailty reflected by upregulated IL−6, TNF‐α and other cytokines—further promotes diaphragmatic protein degradation during the acute phase of ARDS [6]. Third, malnutrition is common among frail patients (as we can note by the very low BMI in this cohort), which additionally restricts substrate availability for required diaphragmatic functional recovery. Collectively, these converging mechanisms render frail patients vulnerable to earlier and more prominent diaphragmatic dysfunction, thus perpetuating a vicious cycle of respiratory muscle failure, dependence on mechanical ventilation, and progressive disuse atrophy.

The supra‐additive synergistic lethal effect found between frailty and diaphragmatic dysfunction in the present study has important clinical implications. At the mechanistic level, frail patients have a global decline in physiological reserve capacity that extends beyond the musculoskeletal system to neurological (cognitive impairment leading to diminished cooperation with respiration), immune (neurological and molecular changes resulting in diminished defenses from infections or enhanced susceptibility for ventilator‐associated pneumonia [VAP] due to poor oral care), and cardiovascular dimensions (impaired interactions between cardiopulmonary systems) [23, 24]. Combined with the increased ventilatory support needs because of diaphragmatic dysfunction, this multilayered reserve depletion produces an exponential effect that far exceeds simple linear summation. From a clinical perspective, this suggests that for ARDS patients with concurrent frailty and diaphragmatic dysfunction, optimizing mechanical ventilation settings alone may not be sufficient to improve prognosis targeted interventions addressing multiple pathophysiological pathways are likely needed. Triaged expiratory muscle impairment co‐existing with inspiratory muscle weakness may make weaning more difficult, and this needs to be examined in future studies [25].

Specific challenges faced in frail ARDS patients while performing perioperative respiratory management. Data from this study show that frail patients were significantly less likely to receive early rehabilitation (33.3% vs. 58.1%) and IMT (27.6% vs. 42.7%) compared with nonfrail patients, probably reflecting clinical caution against aggressive rehabilitation in these most vulnerable of populations. Nevertheless, a growing body of evidence indicates that the administration of correctly dosed IMT is safe and feasible in critically ill patients with advantageous effects on inspiratory muscle strength and functional recovery after extubation [20]. We found that IMT and early rehabilitation reduced mechanical ventilation duration by about 4.2 days in frail patients compared to no intervention, consistent with the notion that targeted respiratory muscle rehabilitation can improve ventilatory function in critically ill patients [20]; increased 28‐day mortality was observed only marginally significantly as sample sizes permitted. This comparison, however, is also subject to indication bias: Treated patients (*n* = 24) were chosen by clinicians to be more hemodynamically stable and compliant—characteristics that remain difficult to capture in propensity models. Subsequent IPTW models revealed a decrease in the MV duration benefit to 3.1 days (95% CI: 0.4–5.8, *p* = 0.024) with a loss of significance for the trend in 28‐day mortality after adjustment (IPTW‐adjusted *p* = 0.198). Consequently, this finding has to be regarded as hypothesis‐generating and a multicenter RCT specifically designed to establish efficacy is needed prior to committing IMT for routine use in this patient population.

This study further affirms the clinical utility of diaphragm ultrasound. Real‐time bedside diaphragm ultrasound in different dimensions of thickness, DTF, and excursion gives additional information to differentiate VIDD from other causes of diaphragmatic dysfunction, which could further aid in mechanical ventilation parameter adjustment like titration of pressure support level based on P0. Excessive inspiratory effort during assisted ventilation causes diaphragm thickening rather than atrophy; thus, monitoring of effort is important [26]. Both baseline DTF and DTF on Days 3 and 7 of mechanical ventilation were closely associated with clinical outcomes in this study, advocating serial diaphragm ultrasound monitoring as a standard of care assessment in ARDS management. Importantly, the baseline diaphragm thickness may also be decreased in patients with ARDS, especially frail individuals, who could relatively lower the predictive value of a threshold < 20% for DTF; thus, an integrated evaluation considering both ΔThickness (the absolute change of diaphragm thickness) and thinning could fit better.

There are some limitations of this study that should be noted: First, the retrospective study design cannot eliminate residual confounding despite adjustment for known confounders by multivariable regression. Second, the use of CFS assessment in the ICU setting is inevitably subjective, and proxy assessments for patients unable to communicate are likely to underestimate frailty severity. Third, the statistical power of subgroup analyses was limited by the relatively small sample size—particularly in the postoperative ARDS subgroup. Fourth, the study was single‐centered at one tertiary center institution (Cangzhou Central Hospital), making it difficult to generalize our findings to other institutions. Fifth, the fact that there were no data on genetic polymorphism and comprehensive proteomic biomarkers hampered an in‐depth mechanistic exploration of the interaction between frailty and diaphragmatic dysfunction. Confirmatory sensitivity analysis (*n* = 148 matched pairs) with PSM found similar results, although residual confounding cannot be ruled out. Seventh, 43.7% of frail patients required proxy CFS assessments (ICC = 0.84, 95% CI: 0.76–0.90); proxy‐based underestimation of frailty severity constitutes thereby a nondifferential bias. Eighth, Benjamini–Hochberg FDR correction applied; weakly powered (*n* < 30) subgroups are considered hypothesis‐generating only. Ninth, clinical utility will require prospective multicenter external validation of the predictive model. Tenth, the IMT subgroup (*n* = 24 treated) is susceptible to indication bias; thus, the IPTW‐adjusted estimate should be considered exploratory pending RCT evidence.

## 5 Conclusions

Prefrailty and diaphragmatic dysfunction are both independent risk factors for which the combination has a synergic effect on mortality in the long term in ARDS. The combined CFS–DTF–APACHE II predictive model (AUC = 0.851–0.889) identified high‐risk patients responsibly well. Perioperative diaphragm ultrasound monitoring, IMT, and early rehabilitation appear to be safe novel strategies that reduce mechanical ventilation duration in frail patients; however, multicenter RCT confirmation of feasibility and effectiveness before routine clinical practice is advised along with external model validation and clinical implementation.

## Author Contributions

Mengmei Ma and Lianchao Men contributed equally as co‐first authors, responsible for study conception, literature review, data collection, and manuscript drafting. Chenhui Fu provided expertise in diaphragmatic ultrasound assessment. Bin Huang assisted with data analysis and institutional coordination. Yu Han participated in data collection and manuscript revision. Danyang Wang, as corresponding author, supervised the research, critically revised the manuscript, and approved the final version. All authors approved the published version.

## Funding

This work was supported by Hebei Province Medical Science Research Project Plan (No. 20251578).

## Ethics Statement

This study was approved by the Ethics Committee of Cangzhou Central Hospital, with approval number 2024‐315‐02.

## Consent

All authors reviewed and approved the final manuscript.

## Conflicts of Interest

The authors declare no conflicts of interest.

## Data Availability

The data can be obtained from the corresponding author for reasonable reasons.
